# Quarantine Department

**Published:** 1900-03

**Authors:** 

**Affiliations:** Secretary Quarantine Department


### QUARANTINE DEPARTMENT.

DR. I. J. JONES, SECRETARY QUARANTINE DEPARTMENT, EDITOR.

The State Health Officer has mailed the following circular letter to the school trustees of the independent school districts of the State. The same letter will be sent to the county judges for distribution to trustees of other districts:

AUSTIN, TEXAS, March 12, 1900.

DEAR SIR: Inclosed please find an opinion by the Attorney-General, setting forth the right of the public school trustees to exclude from attendance all unvaccinated children.

In this connection I desire to urgently represent to you that the smallpox is now epidemic in the United States, and, according to the best judgment of health authorities, will continue to prevail until all the population is vaccinated or has had the disease.

That thorough vaccination will absolutely prevent the disease in nearly every instance, is conclusively proven, and when it fails to prevent an attack, it modifies it into a mild attack of varioloid.

The impression is general that the prevalent smallpox is so mild as to be harmless.

This is true of the disease when it first makes its appearance in a community, but if allowed to spread for any length of time, it assumes a graver type, and already in some communities in this State, and many places in other States, the disease has become extremely malignant and fatal, in some places officially reported as having killed seventy-five per cent. of those attacked.

The opinion inclosed defines your power to enforce vaccination in the schools, a power possessed by no one else, hence you, and you alone, are responsible for the vaccination of the schools. The disease is so widespread that I have no hesitation in saying to you that in the next year or so, and perhaps within a few weeks, smallpox will occur in your community, and if so, and you have failed to secure the vaccination of the schools, you will have neglected a duty that, in the opinion of all health authorities, is imperative.

If you order the schools vaccinated it should be done with strict precautions, insuring the least danger of disagreeable consequences. If every precaution is used in securing good vaccine, and applying it in a cleanly manner, there should be no untoward results of any kind.

The County Health Officer of Cherokee county, last year, vaccinated 800 convicts at one time, with the result that only five were disabled at all, and those were only disabled from work for a day or two each.

I would be glad to hear from you at once to the effect that you had ordered your school vaccinated. If there is any further advice or suggestion you desire, I am at your command.

Your obedient servant,

W. F. BLUNT,
State Health Officer.

